# Histotopographic Reasons for Ventral Approach in Bulbous Non-Transecting Urethroplasty

**DOI:** 10.5152/tud.2025.25003

**Published:** 2025-07-29

**Authors:** Andrey Borisovich Bogdanov, Magomed Islambegovich Katibov, Evgeny Ibadovich, Alexander Evgenievich Sokolov, Inga Vladimirovna Kosova, Vladimir Arshakovich Vardanyan, Yulia Yurievna Andreeva, Olga Alexandrovna Kuznetsova, Genady Inanovich Nichiporuk, Ivan Vasilievich Gayvoronskiy, Badri Roinovich Gvasalia, Francisco Martins, Oleg Borisovich Loran, Dmitry Yurievich Pushkar

**Affiliations:** 1 S.P. Botkin City Clinical Hospital, Moscow, Russia; 2 Russian Medical Academy of Continuous Professional Education, Moscow, Russia; 3 City Clinical Hospital, Makhachkala, Russia; 4 V. P Demihov Moscow City Hospital, Moscow, Russia; 5 S.M. Kirov Military Medical Academy, Saint Petersburg, Russia; 6 Russian University of Medicine, Moscow, Russia; 7 Department of Urology, University of Lisbon, Santa Maria University Hospital, Lisbon, Portugal

**Keywords:** Bulbous urethral stricture, urethroplasty, urethral surgery, excision and primary anastomosis with transection of corpus spongiosum, transecting excision and primary anastomosis, nontransecting excision and primary anastomosis, nontransecting excision and primary anastomosis

## Abstract

**Objective:**

A histotopographic research study was performed to justify the ventral approach for nontransecting anastomotic bulbar urethroplasty.

**Methods:**

The study included 10 preparations of the male penis, including the bulbous sections of the urethra with no signs of structural damage. The material was obtained during autopsy from men aged 36-60 years old. The features of the blood supply and innervation of the bulbous urethra were carefully examined, revealing the advantages of the proposed method.

**Results:**

The authorsobtained data providing sufficient evidence for the safety and histotopographic validity of the ventral approach with preservation of the dorsal and lateral parts of the corpus spongiosum of the urethra.

**Conclusion:**

The choice of urethroplasty for bulbous urethral strictures less than 2 cm in length requires in-depth knowledge of the anatomy of the vascular and autonomic nerve fibers of the spongious body of the bulbous urethra, as well as the course of the cavernous nerves along its dorsal semicircle. In carefully selected patients with short (<2 cm in length) strictures of the bulbous urethra without spongiofibrosis, it is possible to avoid anastomotic urethroplasty with total transection of the spongious body by choosing nontransecting excision and primary anastomosis (ntEPA), which allows to preserve innervation and blood supply in the urethra to a greater extent. In this regard, the ventral ntEPA technique seems promising, as it is likely that the neurovascular structures in the urethra are mostly located outside the area of this zone—in the lateral frequent. However, definitive conclusions are possible after further scientific research in this area.

**Main Points**

- Nontransecting excision and primary anastomosis with ventral approach is the most favorable way of organ-preserving surgical treatment of the short bulbous urethral strictures.
- The most significant blood vessels and nerve fibers in cadaver studies were located at the 3 and 9 o’clock positions on the conventional dial.
- Dorsal mobilization of the proximal part of bulbous urethra carries risk of the cavernous nerve injury.

## Introduction

The surgical treatment of short bulbous urethral strictures is currently clearly regulated with Russian and international recommendations. It includes endoscopic treatment and anastomotic urethroplasty with complete transection of the corpus spongiosum. The latest technique was developed at the beginning of the twentieth century and has been used for 100 years because of its high efficiency and safety.^1^ Among the types of anastomotic surgeries, there is a separate category, specifically surgeries without complete transection of the corpus spongiosum of the urethra, known as nontransecting excision and primary anastomosis (ntEPA) urethroplasty in the international literature. The first publications appeared more than 15 years ago.^2^^-^^5^ Every year, an increasing number of publications appear in the literature that suggest to avoid surgeries with complete urethral transection in individual patients in the anastomotic group.^6^^-^^9^ Both in the case of augmentation urethroplasty, and in the case of ntEPA urethroplasty, a new set of operations has gained popularity, which can be combined with the term “urethra-sparing” or “urethra- preservation,” implying the principle of maximum preservation of the healthy urethral tissue. They are based on the rejection of complete transection of the urethra and sometimes on the complete separation of its posterior semicircle in case of ventral approach to the stricture.^10^^-^^12^ Published data suggest that such a minimally invasive approach is superior in terms of maintaining the continuity of the functional urethra with its vessels and nerves. It is highly likely that this is the reason for the advantages of this approach in sparing sexual function.^8^^,^^13^^,^^14^ Unfortunately, currently there is no single randomized study that directly compares the effectiveness of transecting (tEPA) and ntEPA urethroplasty.^15^ Due to the dissemination of various surgical techniques and the large number of surgeries performed, significant technical experience and an understanding of the biological behavior of urethral tissue have accumulated among the community of urethral surgeons. These concepts have further shifted the interest of the urethral surgeon community toward urethra-saving surgery. However, there is a significant lack of local studies and large fundamental comparative randomized multicenter studies in this area. Nevertheless, the authors expect an increase in the number of such minimally invasive operations in the near future, thanks to individual specialists. Considering the noted trends, despite the fact that the topographic and anatomical features of the bulbous urethra are well studied and described in detail,^16^^,^^17^ it seemed necessary to us to conduct additional research and histotopographically substantiate the new surgical technique to realize the need for the maximum preservation of the functional urethra.

## Material and Methods

**Figure 1.**
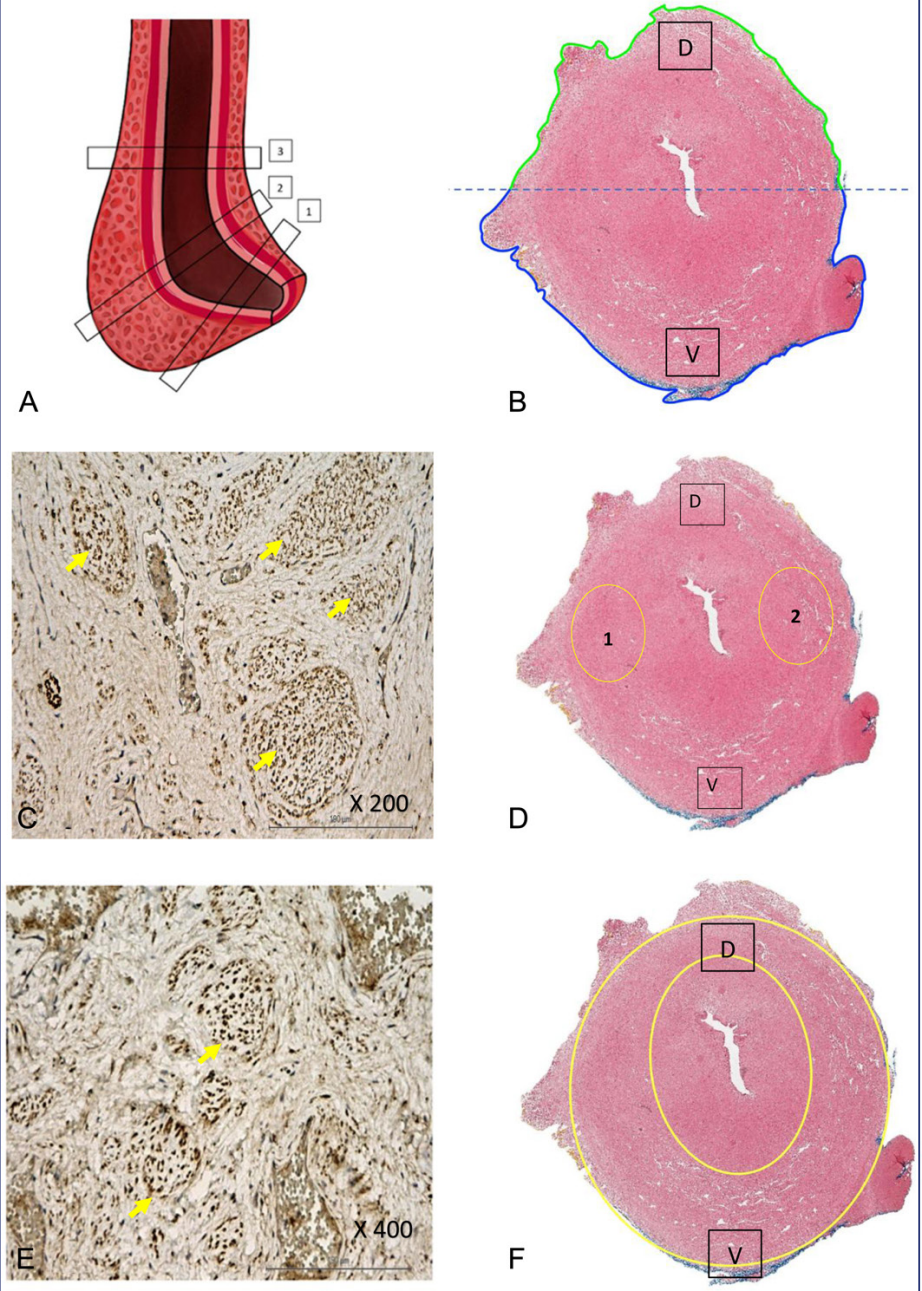
(A) Division of the bulbous urethra into segments: 1—proximal section; 2—middle section; 3—distal section. (B) A cut of the bulbous urethra, magnification 10x, hematoxylin and eosin staining, virtually divided into ventral (marked with blue line and further with letter “V” only) and dorsal (marked with green line and further with letter “D” only) semi-circles. (C) The “largest” nerve fibers (marked with yellow pointers) in the middle segment of a bulbous urethral wall, which (that) mostly located at the 3 and 9 o'clock positions, these areas are marked with yellow circles №1,2 on the picture D (immunohistochemical study in the PGP9, magnification × 200). (D) A cut of the bulbous urethra, magnification 10×, hematoxylin and eosin staining, shows localization of the “largest” nerve fibers presented in picture C (the zones are indicated by the yellow circles with the number 1 on the right and 2 on the left). (E) “Minor” nerve fibers (marked with yellow pointers) are evenly widespread throughout the spongious body (immunohistochemical study of PGP9, magnification × 400). (F) A cut of the bulbous urethra, magnification 10x, hematoxylin and eosin staining, with the localization of “minor” nerve fibers presented in picture E (the whole spongious body is indicated with yellow circle, since “minor” nerve fibers are evenly widespread throughout the spongious body).

The study was approved by S.P. Botkin Municipal Clinical Hospital of Moscow (protocol №7, June 12, 2023). The authors performed an experimental study where 10 preparations of the male penis were evaluated. The bulbous and membranous segments of the urethras were preserved without signs of damage to their structures. The material was obtained during autopsy from men aged 36-60 years old, who did not have diseases of the lower urinary tract. The corpus spongiosum was fixed in 10% neutral buffered formalin for 48 hours. After fixation, the corpus spongiosum was segmented into 3 sections: 1—proximal, 2—middle, and 3—distal (Figure 1A). Then, 2 fragments of the corpus spongiosum were cut out from each surface—ventral semicircle (V) and dorsal semicircle (D), by dividing the material into 2 almost symmetrical halves (Figure 1B).

Using light microscopy, the structure of the urethral epithelium, the structure of the wall of the corpus spongiosum, the vascular channel and nerve fibers were studied at various magnifications (×10, ×100, ×200, ×400). An immunohistochemical study of these areas was also carried out according to standard methods to identify neural and vascular structures.^18^

## Results

Blood vessels were demonstrated in the form of well-defined capillaries along the ventral and dorsal surface of the submucosal layer and the main vessels were based along the lateral surfaces of the corpus spongiosum at the 3 and 9 o’clock positions. The nerve fibers showed a polymorphic structure. Small and scattered nerve fibers were located mainly on the ventral and dorsal surfaces, and larger nerve fibers were significantly located at the 3 and 9 o’clock positions (lateral part of the corpus spongiosum on the right and left) (Figure 1C and D). The medium- and small-sized nerve endings were concentrated around the perimeter and visualized in the dorsal and ventral surfaces of the urethra (Figure 1E and F).

Only in 1 case out of 10 was there a different arrangement of nerve fibers, with a predominance of nerve fibers in the ventral part of the corpus spongiosum.

## Discussion

**Figure 2.**
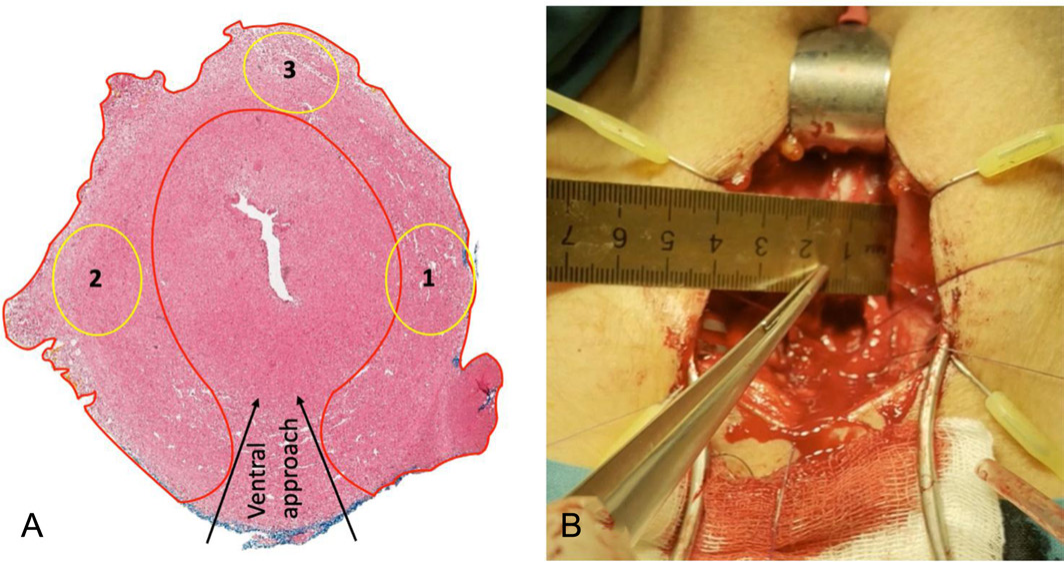
(A) The scheme of approach for ventral anastomotic urethroplasty without transecting the corpus spongiosum.^10^ The boundaries of the red zone indicate the saved corpus spongiosum after the excision of the stricture. Schematic location of the nerve fibers in the preserved lateral and dorsal parts of the corpus spongiosum (yellow circles 1, 2, 3). (B) Intraoperative photo of the preserved lateral part of the corpus spongiosum, the thickness of which was slightly more than 5 mm.

The identified features of the structure and vascular-nerve supply of the bulbous urethra confirm the rationale for choosing the proposed longitudinal ventral approach in surgery without complete transection of the corpus spongiosum, as it preserves the posterior and lateral surfaces of the corpus spongiosum with the nerve fibers and vessels crossing through them (Figure 2). This technique minimizes damage to the innervation and antegrade blood supply of the entire corpus spongiosum.^9^^,^^10^

Other reasons to choose a ventral approach without mobilization of the posterior semicircle of the corpus spongiosum are based on the study of T.F. Lue et al,^1^^8^ in which the location of the cavernous nerve in relation to the membranous and bulbous parts of the urethra was studied. Thus, a possible mechanism for iatrogenic erectile dysfunction during urethral surgery was indicated.^18^

**Figure 3.**
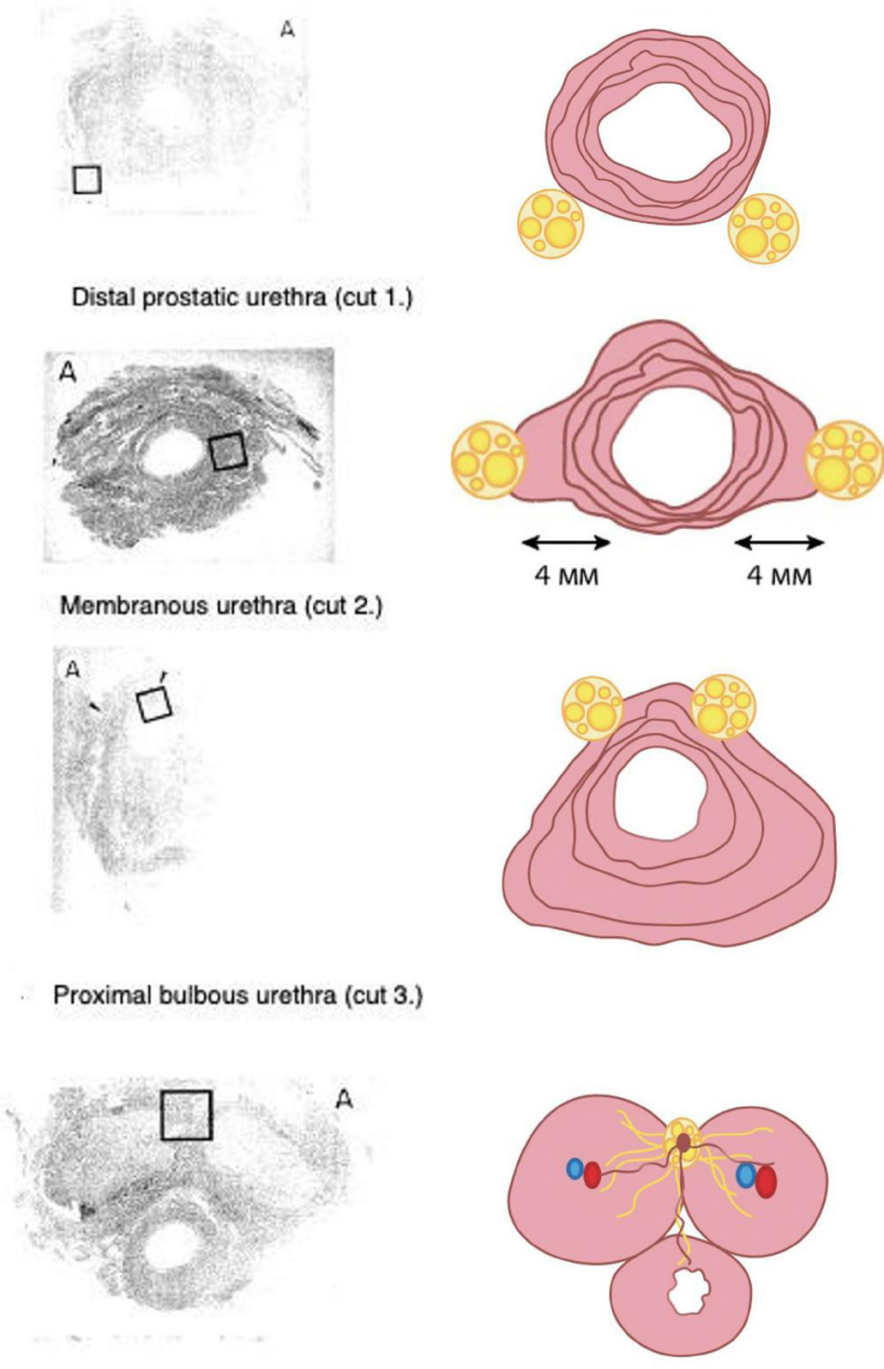
The course of cavernous nerves (yellow) at various levels of the urethra, from the distal prostatic to the distal bulbous area (transverse sections). Column A (left)—the microslide colored hemotoxilin and eosin, magnification× 40 (borrowed from T.F. Lue et al^19^). Column B (right)—the scheme-reconstruction of the microslides of T.F. Lue et al.

**Figure 4.**
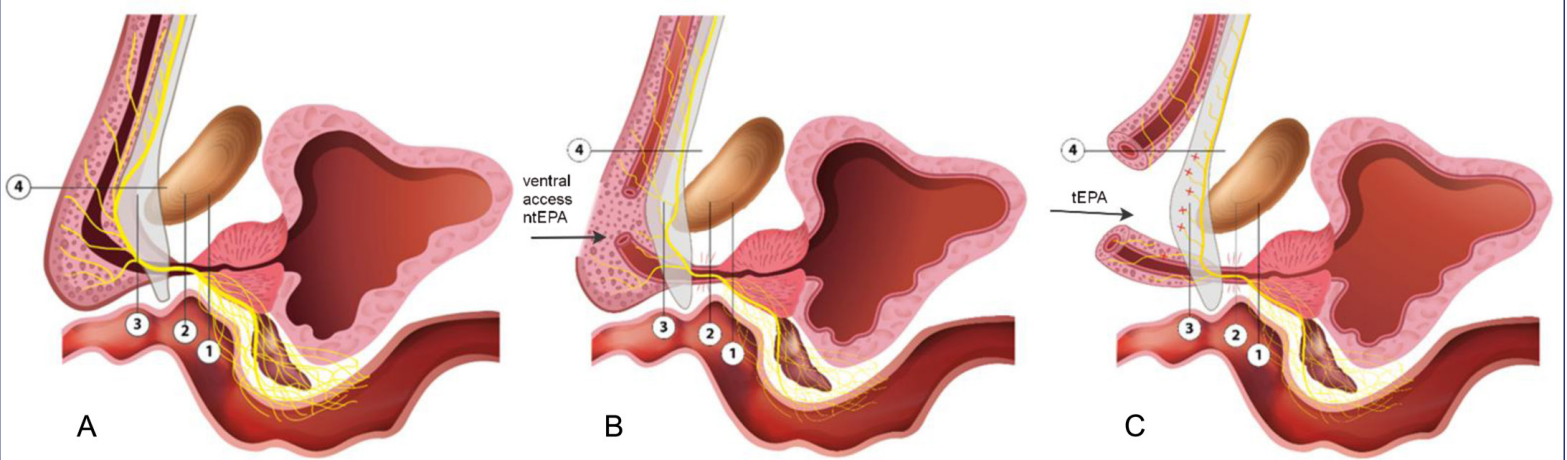
The course of the cavernous nerve and variants of its damage in ventral ntEPA and tEPA. (A) Sagittal cut of the urethra with different levels (1, 2, 3, 4 according to Figure 3) of the cavernous nerve path, reconstruction according to T.F. Lue et al.^19^ (B) Anastomotic urethroplasty without complete transection of the corpus spongiosum (ventral ntEPA). (C) Anastomotic urethroplasty with complete transection of the urethra (tEPA).

Based on the work of T.F. Lue et al,^18^ the authors made a schematic reconstruction of the cavernous nerve path in different parts of the membranous and bulbous urethra, which can improve the understanding of the neuroanatomy of the urethra (Figure 3, on the right side, Figure 4A). According to this study,^18^ the cavernous nerves first follow along the dorsal surface of the distal part of the prostatic urethra (Figure 3, cut 1, Figure 4A), then move to the lateral surface of the membranous urethra (Figure 3, cut 2, Figure 4A), then further to the dorsal surface of the urethra in the area of the proximal part of the bulbous urethra (Figure 3, cut 3, Figure 4A). After they enter the intra-cavernous space in the distal part of the bulbous urethra, they follow along the dorsal surface of the corpus cavernosum (Figure 3, cut 4, Figure 4A).

Using this scheme, you can better understand the potential advantages of the surgical urethroplasty technique without complete transection of the corpus spongiosum (Figure 4).

The cavernous nerves are represented by 2 types of fibers, innervating the corpus cavernosum and the corpus spongiosum of the urethra. In another study B. Alsaid et al^19^, using immunohistochemical studies, investigated the anatomical and topographic features of the prostatic nervous plexus forming the cavernous nerve.^19^ Their data are similar to the results of the authors’ work.

Thus, the cavernous nerve not only forms the main efferent nerve fibers going to the corpus cavernosum but also gives off some branches to the corpus spongiosum, which can be responsible for its autonomic innervation.

Due to the understanding of this arrangement of the cavernous nerves and using this knowledge during radical prostatectomy, functional outcomes in patients with erectile function and continence have been significantly improved. Unfortunately, these fundamental principles are only beginning to be actively applied in urethral surgery. Considering all the data presented, it should be recognized that the ventral approach in anastomotic urethroplasty without complete transection of the urethra seems to be a fairly reasonable and potentially beneficial technique in many respects. These facts should help overcome certain existing preconceived ideas that anastomotic urethroplasty without complete transection of the urethra has a greater number of relapses and complications. Much of the research conducted in recent years underscores this point. For example, a review by G. Barbagli et al^1^ compared techniques with urethral transection, which included the anastomotic urethroplasty technique with complete transection and the augmentation anastomotic technique (roof-strip technique), as well as techniques without transection, which included anastomotic urethroplasty without complete transection and augmentation techniques using the buccal mucosa grafting. Treatment success was comparable and amounted to 90-98.6% and 81.8-100%, respectively.^20^ Thus, it has become clear that the skepticism in actively implementing anastomotic urethroplasty techniques without complete transection is not critical, but rather reflects a sort of surgeon’s bias. Major rethinking of new approaches and their adoption by the urological community is urgently needed. Therefore, in the authors’ opinion, any research in this area is highly needed and should be pursued.

The selection of the urethroplasty technique for bulbous urethral strictures less than 2 cm requires an in-depth knowledge of the vascular and autonomic nerve fiber anatomy of the corpus spongiosum of the bulbous urethra as well as the course of the cavernous nerves along its dorsal semi-circle. The ventral approach clearly shows that it is advisable to abandon the anastomotic urethroplasty with total transection of the corpus spongiosum in carefully selected patients with short (<2 cm in length) strictures of the bulbous urethra without spongiofibrosis. In the authors’ opinion, ntEPA by ventral longitudinal approach is the most favorable surgical treatment option in these patients, as it is associated with optimal preservation of the innervation and blood supply on the dorsal and lateral aspects of the preserved corpus spongiosum of the urethra.

## Data Availability

The data that support the findings of this study are available on request from the corresponding author.
